# Depression and anxiety in cervical degenerative disc disease: Who are susceptible?

**DOI:** 10.3389/fpubh.2022.1002837

**Published:** 2023-01-06

**Authors:** Dacheng Sang, Bowei Xiao, Tianhua Rong, Bingxuan Wu, Wei Cui, Jianhao Zhang, Yue Zhang, Baoge Liu

**Affiliations:** Department of Orthopaedic Surgery, Beijing Tiantan Hospital, Capital Medical University, Beijing, China

**Keywords:** anxiety, cervical degenerative disc disease, cervical spine, depression, mental health disorders

## Abstract

**Background:**

Pre-operative depression and anxiety are associated with poorer patient-reported outcomes following cervical spine surgery. Identification of and interventions for these disorders are key to preventing related negative effects. However, most spine surgeons do not routinely evaluate mental health disorders. Few studies have investigated which patients with cervical degenerative disc diseases (CDDD) are susceptible to depression and anxiety.

**Objective:**

To determine the factors associated with depression and anxiety in patients with CDDD.

**Methods:**

Three hundred twelve patients with CDDD were recruited in this cross-sectional case-control study. Patients underwent a structured interview to acquire demographic and clinical characteristic information, which included the Neck Disability Index (NDI), modified Japanese Orthopedic Association (mJOA), and Visual Analog Scale (VAS) for neck/arm pain. Depression and anxiety were evaluated using the Zung Self-Rating Depression and Anxiety Scales. Univariate and multivariate logistic regression analyses were used to identify factors associated with depression and anxiety.

**Results:**

Of all patients, 102 (32.7%) had depression and 92 (29.5%) had anxiety. Two hundred six (66.0%) patients with neither depression nor anxiety were defined as the control group. Univariate analysis indicated that gender, educational level, occupation type, Charlson comorbidity index, symptom duration, symptomatology, surgery history, NDI, mJOA, VAS-neck, and VAS-arm scores were associated with depression and anxiety (except for symptom duration for anxiety). Multivariate logistic regression analysis indicated that females [odds ratio (OR) 1.81, 95% confidence interval (CI) 1.01–3.23], physical work (OR 2.06, 95% CI 1.16–3.65), poor mJOA score (OR_moderate_ 2.67, 95% CI 1.40–5.07; OR_severe_ 7.63, 95% CI 3.85–15.11), and high VAS-neck score (OR 1.24, 95% CI 1.11–1.39) were independent risk factors for depression. Physical work (OR 1.84, 95% CI 1.01–3.35), poor mJOA score (OR_moderate_ 2.66, 95% CI 1.33–5.33; OR_severe_ 9.26, 95% CI 4.52–18.99), and high VAS-neck score (OR 1.34, 95% CI 1.19–1.51) were independent risk factors for anxiety.

**Conclusion:**

Approximately one-third of patients with CDDD had depression or anxiety. Patients who engaged in heavy work and had severe symptoms (poor mJOA and high VAS-neck scores) are susceptible to depression and anxiety. Additionally, female patients are susceptible to depression. Our findings may help identify CDDD patients with depression and anxiety in clinical practice.

## Introduction

Cervical degenerative disc disease (CDDD) is one of the most common disorders that leads to neck pain, cervical radiculopathy, and/or myelopathy (1). Neck pain is a multifactorial disease, and one of the most common and obvious complaints of patients with CDDD (2–4). Reportedly, neck pain was ranked the fourth leading cause of disability and it affected nearly 15% of the global population (5, 6). Total annual treatment costs for neck pain were estimated at $686 million in Netherlands and $800 million in China (5, 7). Currently, cervical spine surgery is widely accepted as the standard treatment for patients with CDDD and offers significant improvements in patient-reported outcomes (8). However, not all patients experience significant improvements in pain or disability following cervical spine surgery. Several preoperative factors, such as depression and anxiety, have been reported to affect surgical outcomes (9, 10).

Numerous studies have indicated that preoperative depression and anxiety affect surgical outcomes and patient satisfaction (11–17). Unrecognized effects and inappropriate interventions for preoperative depression and anxiety may significantly negatively affect patient-reported outcomes (18). Furthermore, depression and anxiety have been reported as risk factors for complications, readmission, and revision following cervical spine surgery (19). Besides, mental stress related symptoms, including depression and anxiety, are associated with changes in masticatory muscle symmetry, indicating that mental state is related to muscle function (20). Therefore, it is beneficial for spine surgeons to identify CDDD patients who are susceptible to depression and anxiety. Jablonska et al. (21) investigated the demographic factors associated with depression in patients with cervical or lumbar degenerative disc disease. Additionally, Stoffman et al. (18) studied the relationship between the severity of cervical myelopathy and depression/anxiety based on a relatively small cohort. To date, there are little specific attention paid to the factors related to pre-operative depression and anxiety in CDDD patients. Moreover, the negative effects of depression and anxiety on surgery outcomes may currently be underestimated, since mental health status is not evaluated routinely in clinical practice. Generally, patient demographics and clinical characteristics, such as the Neck Disability Index (NDI), modified Japanese Orthopedic Association (mJOA), and Visual Analog Scale (VAS) scores are routinely assessed and recorded. Therefore, it is essential for spine surgeons to understand the relevant factors from the demographics and clinical characteristics of patients to predict which patients are susceptible to depression or anxiety.

This study aimed to determine the factors associated with depression and anxiety in CDDD patients. According to previous studies and our clinical experience, we hypothesized that disease severity is associated with depression and anxiety in CDDD patients; patients with poor mJOA and high VAS scores are more susceptible to depression and anxiety.

## Methods

### Study population

All patients (*n* = 346) who underwent cervical spine surgery for CDDD at our institution between January 2019 and June 2021 were invited to participate in this cross-sectional case-control study. Nine patients declined participation and 25 patients did not provide complete information. Therefore, 312 patients with CDDD (effective response rate: 90.2%) were finally recruited in this study. All patients were older than 18 years, presented with typical symptoms and signs of CDDD, and showed no improvement after at least 3 months of conservative treatment. Typical symptoms and signs of CDDD may present with neck pain, radiculopathy (radicular arm pain, paresthesia, numbness, or weakness), myelopathy (spinal cord dysfunction and upper motor neuron impairment), or a combination of these symptoms (1, 22). Patients with non-degenerative (e.g., trauma, tumor, and infection) or neuromuscular diseases (e.g., motor neuron disease) were excluded. Informed consent was obtained from all patients. The study design was approved by the Ethics Committee of Beijing Tiantan Hospital, Capital Medical University (Ethical Approval Number: KY 2020-073-02).

### Data collection

Patient demographics including age, gender, body mass index (BMI), occupation type, educational level, smoking and drinking history, hypertension, diabetes mellitus, cardiovascular diseases, cerebrovascular diseases, and Charlson Comorbidity Index (CCI) (23) were recorded in detail. Clinical data comprising preoperative symptomatology, symptom duration, and history of cervical spine surgery were also recorded. Patient-reported outcomes were evaluated during the week before surgery using the following clinical outcome measures: mJOA score, NDI, and 10-point VAS-neck and VAS-arm.

The mJOA score and NDI are two measures that assess the severity of myelopathy symptoms and disability for patients with CDDD (24). According to the literature, mild myelopathy is defined as an mJOA score of 15–17, moderate as an mJOA score from 12 to 14, and severe as an mJOA score of 11 or less (25). NDI scores can be categorized as no disability ( ≤ 8%), mild disability (10–28%), moderate disability (30–48%), severe disability (50–68%), and complete disability (≥70%) (26, 27).

Depression and anxiety were evaluated using the Zung Self-Rating Depression Scale (SDS) (28) and the Zung Self-Rating Anxiety Scale (SAS) (29). The SDS and SAS have been validated as acceptable screening tools for assessing the level of depression and anxiety, respectively (16). SDS and SAS scores of 50 or higher indicate the presence of depression and anxiety, respectively (30).

### Statistical analysis

Statistical analyses were conducted using SPSS 23.0 software (IBM Corp., Armonk, NY, USA) by an investigator who was not involved in the data collection. PASS 15.0.5 software (NCSS, LLC, USA) was used to calculate sample size. Shapiro–Wilk test was performed to identify whether continuous data conform to a normal distribution (*p* > 0.05). Normally distributed continuous variables are presented as means ± standard deviations (SD) and were compared using independent samples *t*-tests. Non-parametric variables are presented as medians and ranges (25th and 75th percentiles) and were compared using the Mann-Whitney *U*-test. Categorical variables are presented as frequencies and percentages and were compared using the chi-square test. Bivariate correlations between patient demographic and clinical characteristics were explored using Kendall's tau-b analysis because several variables were categorical, such as gender, educational level, and occupation type. Odds ratios (ORs) and 95% confidence intervals (CIs) for independent risk factors of depression and anxiety in patients with CDDD were calculated using multivariate logistic regression analysis. Variables that had a *p* < 0.1 in the univariate analysis were controlled in the logistic regression analysis. Tolerance and variance inflation factor (VIF) were calculated to identify potentially multicollinear variables. To address issues of multicollinearity, a stepwise regression method was used to screen out independent variables. Statistical significance was set at *p* < 0.05.

## Results

### Patient demographic and clinical characteristics

**Figure 1:**
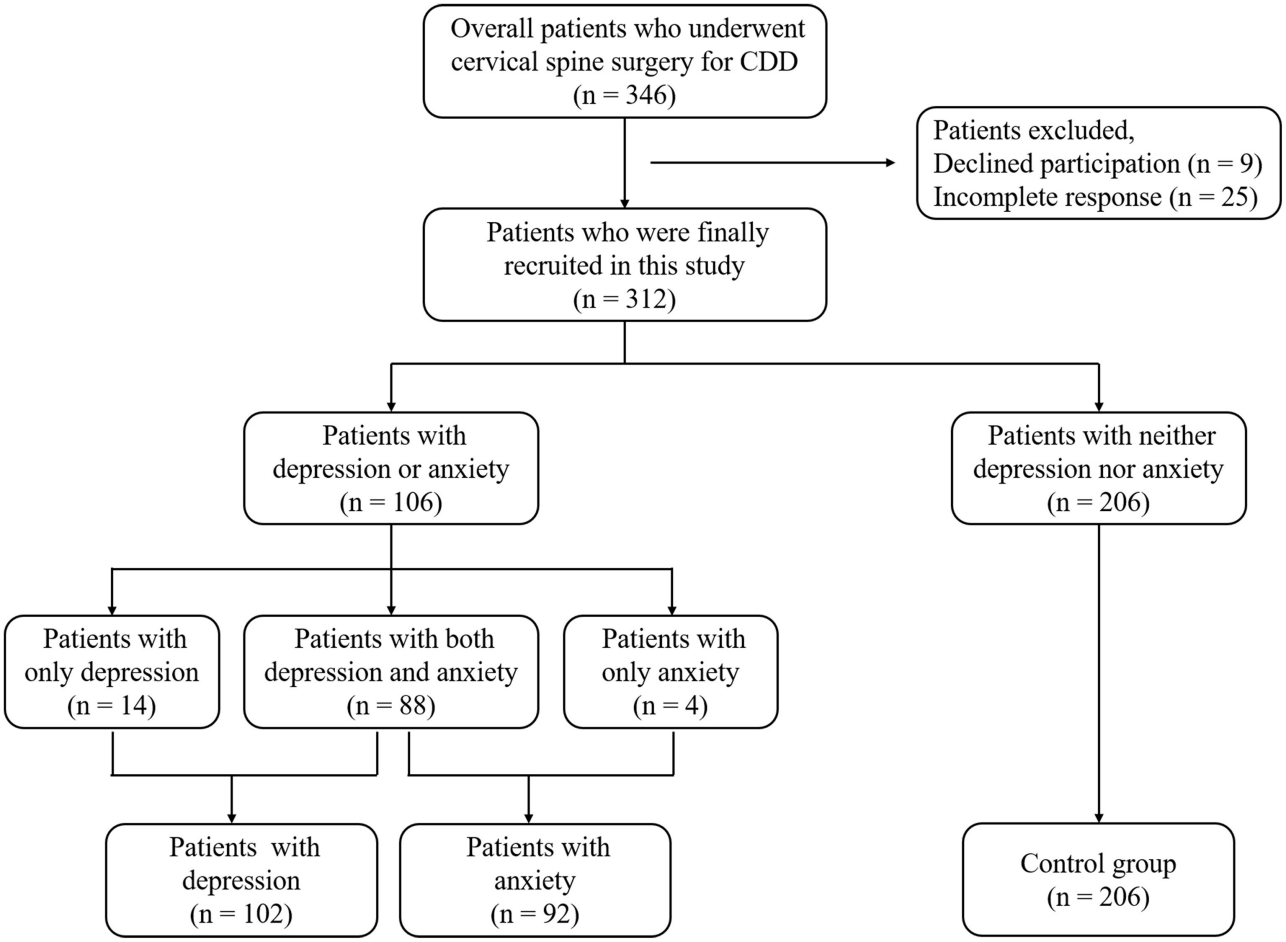
Flowchart of the study population.

Of the 312 CDDD patients (182 males and 130 females, mean age 57.9 ± 9.8 years), 102 patients (32.7%) had depression, 92 patients (29.5%) had anxiety, and 88 patients (28.2%) had both depression and anxiety. Patients with neither depression nor anxiety (*n* = 206, 66.0%) were defined as the control group (Figure 1). Description and comparison of patient demographic and clinical characteristics are shown in Table 1.

**Table 1:** Description and comparison of patient demographic and clinical characteristics.

	**Depression cases and controls**			**Anxiety cases and controls**		

	**Depression** **(*****n*** = **102)**	**Controls** **(*****n*** = **206)**	***P***	**Anxiety** **(*****n*** = **92)**	**Controls** **(*****n*** = **206)**	***P***
Age (ye)	58.7 ± 9.0	57.4 ± 10.1	0.263	58.6 ± 9.4	57.4 ± 10.1	0.368
Gender			**0.034**			**0.027**
Male [*n* (%)]	51 (50.0)	129 (62.6)		45 (48.9)	129 (62.6)	
Female [*n* (%)]	51 (50.0)	77 (37.4)		47 (51.1)	77 (37.4)	
Educational level			**0.017**			**0.047**
Primary [*n* (%)]	29 (28.4)	32 (15.5)		25 (27.2)	32 (15.5)	
Junior [*n* (%)]	34 (33.3)	64 (31.1)		31 (33.7)	64 (31.1)	
Senior [*n* (%)]	30 (29.4)	74 (35.9)		27 (29.3)	74 (35.9)	
University [*n* (%)]	9 (8.8)	36 (17.5)		9 (9.8)	36 (17.5)	
Occupation type			**< 0.001**			**< 0.001**
Intellectual [*n* (%)]	28 (27.5)	106 (51.5)		25 (27.2)	106 (51.5)	
Physical [*n* (%)]	74 (72.5)	100 (48.5)		67 (72.8)	100 (48.5)	
Hypertension [*n* (%)]	53 (52.0)	85 (41.3)	0.076	47 (51.1)	85 (41.3)	0.115
Diabetes mellitus [*n* (%)]	28 (27.5)	41 (19.5)	0.135	27 (29.3)	41 (19.5)	0.073
Cardiovascular disease [*n* (%)]	12 (11.8)	19 (9.2)	0.487	10 (10.9)	19 (9.2)	0.658
Cerebrovascular diseases [*n* (%)]	16 (15.7)	24 (11.4)	0.321	15 (16.3)	24 (11.4)	0.271
CCI^‡^	2 (1, 3)	1 (0, 2)	**0.002**	2 (1, 3)	1 (0, 2)	**0.004**
CCI (category)			**0.004**			**0.004**
≤ 1 point	48 (47.1)	126 (61.2)		44 (47.8)	126 (61.2)	
2 points	19 (18.6)	44 (21.4)		16 (17.4)	44 (21.4)	
≥3 points	35 (34.3)	36 (17.5)		32 (34.8)	36 (17.5)	
BMI (kg/m^2^)	25.2 ± 3.3	25.5 ± 3.5	0.551	25.1 ± 3.4	25.5 ± 3.5	0.454
BMI (WHO-Asian category)			0.770			0.772
Normal [*n* (%)]	29 (28.4)	51 (24.8)		27 (29.3)	51 (24.8)	
Overweight [*n* (%)]	47 (46.1)	102 (49.5)		42 (45.7)	102 (49.5)	
Obese [*n* (%)]	26 (25.5)	53 (25.7)		23 (25.0)	53 (25.7)	
Smoke [*n* (%)]	30 (29.4)	65 (31.6)	0.702	28 (30.4)	65 (31.6)	0.847
Drink [*n* (%)]	24 (23.5)	51 (24.3)	0.813	19 (20.7)	51 (24.3)	0.440
Symptom duration (mo)^‡^	30 (5, 72)	12 (4, 48)	0.155	24 (4.5, 66)	12 (4, 48)	0.290
Symptom duration (category)			**0.039**			0.111
< 1 year	43 (42.2)	108 (52.4)		39 (42.4)	108 (52.4)	
1–5 years	31 (30.4)	66 (32.1)		30 (32.6)	66 (32.1)	
>5 years	28 (27.5)	32 (15.5)		23 (25.0)	32 (15.5)	
Pre-operative symptomatology			**0.001**			**0.002**
Radiculopathy [*n* (%)]	14 (13.7)	60 (29.1)		13 (14.1)	60 (29.1)	
Myelopathy [*n* (%)]	38 (37.7)	83 (40.3)		33 (35.9)	83 (40.3)	
Myeloradiculopathy [*n* (%)]	50 (49.0)	63 (30.6)		46 (50.0)	63 (30.6)	
Surgery history			**0.032**			**0.016**
Primary surgery [*n* (%)]	91 (89.2)	197 (95.6)		81 (88.0)	197 (95.6)	
Revision surgery [*n* (%)]	11 (10.8)	9 (4.4)		11 (12.0)	9 (4.4)	
NDI^‡^	29 (10.0, 42.0)	17 (8.0, 29.5)	**< 0.001**	30 (14.0, 44.0)	17 (8.0, 29.5)	**< 0.001**
NDI (category)			**< 0.001**			**< 0.001**
No disability [*n* (%)]	25 (24.5)	67 (32.5)		21 (22.8)	67 (32.5)	
Mild disability [*n* (%)]	26 (25.5)	89 (43.2)		22 (23.9)	89 (43.2)	
Moderate disability [*n* (%)]	33 (32.4)	41 (19.9)		32 (34.8)	41 (19.9)	
Severe disability [*n* (%)]	18 (17.6)	9 (4.4)		17 (18.5)	9 (4.4)	
mJOA^‡^	12 (10.5, 14.0)	14 (13.0, 15.5)	**< 0.001**	12 (10.0, 14.0)	14 (13.0, 15.5)	**< 0.001**
mJOA (category)			**< 0.001**			**< 0.001**
Mild myelopathy [*n* (%)]	17 (16.7)	93 (45.1)		14 (15.2)	93 (45.1)	
Moderate myelopathy [*n* (%)]	39 (38.2)	80 (38.8)		32 (34.8)	80 (38.8)	
Severe myelopathy [*n* (%)]	46 (45.1)	33 (16.0)		46 (50.0)	33 (16.0)	
VAS-neck^‡^	4 (0, 6)	2 (0, 4)	**< 0.001**	4 (1, 6)	2 (0, 4)	**< 0.001**
VAS-arm^‡^	4 (0, 6)	3 (0, 6)	**0.020**	4 (0, 6)	3 (0, 6)	**0.017**

Data are expressed as either the mean value ± standard deviation, median and range (25th and 75th percentiles), or frequency (%) of patients. Independent sample t-tests, Mann-Whitney U-tests, or chi-square tests were used for univariate analyses. CCI, Charlson comorbidity index; BMI, body mass index; NDI, Neck Disability Index; mJOA, modified Japanese Orthopedic Association score; VAS, Visual Analog Scale; SDS, Self-Rating Depression Scale; SAS, Self-Rating Anxiety Scale; SF-36, 36-item Short-form Health Survey; PCS, physical component summary score; MCS, mental component summary score.^‡^Non-parametric variables. The bold values indicate the values of *p* < 0.05.

There were significant differences between the depression and control groups in gender, educational level, occupation type, and CCI category (all *p* < 0.05). Significant differences were also found between the two groups for symptom duration, symptomatology, surgery history, NDI category, mJOA score category, VAS-neck score, and VAS-arm score (all *p* < 0.05). However, there were no significant differences in age, hypertension, diabetes mellitus, cardiovascular disease, cerebrovascular diseases, BMI, smoking, or drinking between the depression and control groups (all *p* > 0.05).

Between the anxiety and control groups, there were significant differences in gender, educational level, occupation type, diabetes mellitus, and CCI category (all *p* < 0.05). Significant differences between anxiety and non-anxiety groups were also found in symptomatology, surgery history, NDI category, mJOA category, VAS-neck score, and VAS-arm score (all *p* < 0.05). However, there were no significant differences between anxiety and control groups in age, hypertension, cardiovascular disease, cerebrovascular diseases, BMI, smoking, drinking, and symptom duration (all *p* > 0.05).

### Correlation analysis between variables

Bivariate correlations between patient demographic and clinical characteristics are detailed in Table 2. A significant association was found between educational level and occupation type (*r* = −0.439, *p* < 0.001). It should be noted that the mJOA score was significantly associated with several variables, which included gender, educational level, occupation type, CCI, symptom duration, preoperative symptomatology, surgery history, VAS-neck score, and VAS-arm score (all *p* < 0.05). Furthermore, there were significant associations among NDI score, VAS-neck score, and VAS-arm score (all *p* < 0.001).

**Table 2:** Bivariate correlations between patient demographic and clinical characteristics.

	**Gender^#^**	**Educational level^#^**	**Occupation type^#^**	**CCI^#^**	**Symptom duration^#^**	**Symptom-atology^#^**	**Surgery history^#^**	**NDI^#^**	**mJOA^#^**	**VAS-neck**	**VAS-arm**	**Depression^#^**	**Anxiety^#^**
Gender^#^	1												
Educational level^#^	−0.094	1											
Occupation type^#^	−0.07	−0.439^**^	1										
CCI^#^	0.062	−0.174^**^	0.157^**^	1									
Symptom duration^#^	−0.044	−0.081	0.077	0.024	1								
Symptomatology^#^	−0.007	−0.079	0.128^*^	0.171^**^	0.088	1							
Surgery history^#^	−0.062	−0.021	−0.007	0.031	0.246^**^	0.063	1						
NDI^#^	0.238^**^	−0.088	0.09	0.064	0.024	0.181^**^	0.013	1					
mJOA^#^	−0.113^*^	−0.138^**^	0.236^**^	0.130^*^	0.172^**^	0.409^**^	0.219^**^	−0.078	1				
VAS-neck	0.280^**^	−0.086	0.085	0.041	0.026	0.136^**^	−0.045	0.678^**^	−0.101^*^	1			
VAS-arm	0.189^**^	−0.07	0.021	0.06	−0.009	0.125^**^	−0.07	0.685^**^	−0.170^**^	0.496^**^	1		
Depression^#^	0.118^*^	−0.164^**^	0.227^**^	0.164^**^	0.125^*^	0.192^**^	0.124^*^	0.197^**^	0.316^**^	0.175^**^	0.117^*^	1	
Anxiety^#^	0.119	−0.133^*^	0.230^**^	0.170^**^	0.096	0.188^**^	0.140^**^	0.235^**^	0.324^**^	0.227^**^	0.145^*^	0.890^**^	1
SF-36 PCS	−0.017	0.010	−0.09	−0.101^*^	−0.083	−0.238^**^	−0.101^*^	−0.239^**^	−0.287^**^	−0.193^**^	−0.150^**^	−0.325^**^	−0.327^**^
SF-36 MCS	−0.081	0.106^*^	−0.202^**^	−0.112^*^	−0.078	−0.132^**^	−0.091	−0.201^**^	−0.192^**^	−0.216^**^	−0.132^**^	−0.587^**^	−0.559^**^

CCI, Charlson comorbidity index; NDI, Neck Disability Index; mJOA, modified Japanese Orthopedic Association score; VAS, Visual Analog Scale; SF-36, 36-item Short-form Health Survey; PCS, physical component summary score; MCS, mental component summary score. ^#^Categorical variables; ^*^p < 0.05; ^**^p < 0.001.

### Multivariate logistic regression analysis for patients

No significant multicollinearity was found between variables (all tolerance < 0.2 and VIF > 5). Multivariate regression analysis to assess associations between demographic and clinical characteristics and depression or anxiety in CDDD patients, adjusted for possible confounders, identified that females [odds ratio (OR) 1.81, 95% confidence interval (CI) 1.01–3.23], physical work (OR 2.06, 95% CI 1.16–3.65), poor mJOA score (OR_moderate_ 2.67, 95% CI 1.40–5.07; OR_severe_ 7.63, 95% CI 3.85–15.11), and high VAS-neck score (OR 1.24, 95% CI 1.11–1.39) were independent factors associated with depression (Table 3, all *p* < 0.05). Furthermore, physical work (OR 1.84, 95% CI 1.01–3.35), poor mJOA score (OR_moderate_ 2.66, 95% CI 1.33–5.33; OR_severe_ 9.26, 95% CI 4.52–18.99), and high VAS-neck score (OR 1.34, 95% CI 1.19–1.51) were independent factors associated with anxiety (Table 3, all *p* < 0.05).

**Table 3:** Multivariate logistic regression analysis of patient characteristics.

	**Depression**		**Anxiety**	

	**OR (95% CI)**	***P-*** **value**	**OR (95% CI)**	***P-*** **value**
**Gender**				
Male	1		–	–
Female	1.81 (1.01–3.23)	0.045		
**Occupation type**				
Intellectual	1		1	
Physical	2.06 (1.16–3.65)	0.014	1.84 (1.01–3.35)	0.046
**mJOA**				
Mild myelopathy	1		1	
Moderate myelopathy	2.67 (1.40–5.07)	0.003	2.66 (1.33–5.33)	0.006
Severe myelopathy	7.63 (3.85–15.11)	< 0.001	9.26 (4.52–18.99)	< 0.001
VAS-neck	1.24 (1.11–1.39)	< 0.001	1.34 (1.19–1.51)	< 0.001

mJOA, modified Japanese Orthopedic Association score; VAS, Visual Analog Scale; OR, odds ratio; CI, confidence interval.

## Discussion

Depression and anxiety have been reported as common comorbid mental health disorders in CDDD patients. Previous studies have demonstrated a strong association between depression/anxiety and poorer clinical outcomes following cervical spine surgery (11–17). Our results showed that the prevalence of depression and anxiety among CDDD patients was 32.7 and 29.5%, respectively, which is consistent with several previous studies (31). The prevalence of depression (ranging from 23.9 to 43.0%) and anxiety (ranging from 24.7 to 45.6%) in CDDD patients varies significantly between studies (12, 16, 32–35). Differences in study populations and assessment scales may contribute to this inconsistency. Nevertheless, the prevalence of depression and anxiety in CDDD patients is substantially higher than that in the general population (36–38). Given such high prevalence rates and the closeness of the relationships between depression/anxiety and poorer surgical outcomes in CDDD patients (11–17), more attention should be paid to the mental health of CDDD patients in clinical practice.

The results of this study confirmed our hypothesis that disease severity is associated with depression and anxiety in patients with CDDD. The univariate analysis showed that high NDI score, poor mJOA score, and high VAS-neck and VAS-arm scores were risk factors for depression and anxiety, which suggested that patients with greater dysfunction in daily life, more severe myelopathy and pain are more susceptible to depression or anxiety. This result is in line with previous studies (5, 13, 33, 39, 40). Levin et al. (33) found that depressed patients have higher preoperative neck pain scores. In addition, another study found that depressed patients have significantly more baseline pain and disability than those of non-depression patients, as measured by NDI, VAS-neck, and VAS-arm scores (39). Similarly, patients who had a mental health disorder before cervical spine surgery have significantly higher NDI and VAS-neck scores and lower mJOA scores than those who didn't (40). It is worth noting that the objective of these studies was to investigate the association between preoperative depression/anxiety and postoperative clinical outcomes rather than to identify risk factors for depression and anxiety. Thus, we designed this cross-sectional case-control study in a relatively large population to determine the preoperative factors associated with depression and anxiety in CDDD patients. We observed significant differences in NDI, mJOA, VAS-neck, and VAS-arm scores between depression and control groups and between anxiety and control groups. Furthermore, the multivariate regression analysis revealed that mJOA and VAS-neck scores were independent factors for depression and anxiety in CDDD patients. The risk of depression and anxiety in patients with moderate myelopathy was 1.67 and 1.66 times higher than that in patients with mild myelopathy, respectively. In patients with severe myelopathy, the risk of depression and anxiety increased to 6.63 times and 8.26 times, respectively. For each 1-point increase in VAS-neck, the risk of developing depression and anxiety increased by 24 and 34%, respectively. This suggests that patients who had severe symptoms (poor mJOA and high VAS-neck scores) are susceptible to depression and anxiety. Long-lasting pain and disability caused by CDDD can be a mental burden to patients and can affect psychological/psychosocial situation, which promote them to developing depression and/or anxiety. This may be the reason for CDDD patients with high VAS-neck and poor mJOA scores susceptible to depression and anxiety. In contrast, anxiety and depressive disorders can affect patient perception of and susceptibility to pain and disability. Severe symptoms of CDDD patients and poor mental health situation interact with each other and form a vicious cycle.

In the present study, the univariate analysis showed that the female gender was significantly associated with depression and anxiety in CDDD patients. Logistic regression analysis indicated that gender was an independent factor for depression. Female CDDD patients had an 81% increased risk of depression compared with male patients, which suggests that female patients are more susceptible to depression. This finding is supported by a longitudinal observational study that revealed that women who underwent cervical or lumbar disc surgery exhibited significantly more symptoms of depression during the preoperative period (31). This gender difference in mental condition was also confirmed in patients with lumbar degenerative disc disease and the general population (21, 41, 42). One possible explanation for this finding is the higher sensitivity to disability and pain in female patients; when experiencing severe disability and pain, female patients may experience more depression than do male patients. The difference in prevalence of depression between men and women is thought to be linked to individual differences in susceptibility in terms of biological and psychological factors, and environmental factors that operate on both micro and macro levels (43).

CDDD patients with a lower educational level who engaged in physical work were also at higher risk of developing depression and anxiety. Compared with patients who engaged in intellectual work, those who engaged in physical work had a 1.06- and 0.84-fold increased risk of depression and anxiety, respectively. We found a significant negative correlation between educational level and occupation type, which indicated that patients with a lower educational level work in professions that involve heavier physical labor. Recent studies have demonstrated that patients with degenerative cervical and lumbar diseases who had a lower education level were at a greater risk of developing depression and anxiety (21, 31). Jablonska et al. (21) found that unemployment and standing at work were risk factors for depression in patients with cervical and lumbar disc disease, respectively. The physical demands of heavy work professions are significantly higher than those of intellectual professions, which may lead to concerns about persisting health problems (31). Moreover, the deterioration of economic status following sickness absence or decreased working competence due to disability and pain may be more problematic in those who engaged in physical work than in patients who are in mental labor professions (21). Previous studies have highlighted that the unmet needs for individual information on the diagnosis and treatment of disease is associated with depression and anxiety (44). However, patients with a lower educational level or those who are engaged in physical work may have limited ability to acquire such information, which may further promote the deterioration of their mental health.

It is notable that although both educational level and occupation type were identified as risk factors for depression and anxiety, the multivariate logistic regression analysis revealed that only occupation type was an independent risk factor for depression and anxiety in CDDD patients. The bivariate correlation analysis showed a significant association between educational level and occupation type (Table 2). The effect of educational level on depression and anxiety may be influenced by occupation type, and the univariate analysis showed that the effect of occupation type was greater than that of educational level. Therefore, occupation type, rather than educational level, was kept in the final model following stepwise regression analysis.

Previous studies have provided conflicting evidence on whether age affects the mental health of CDDD patients (31, 33). Levin et al. (33) reported that younger patients were more susceptible to developing depression, whereas Lobner et al. (31) found that older age was a significant risk factor for depression. We did not find a significant association between age and depression or anxiety. This result is consistent with a recent review that revealed that the prevalence of depression in patients with degenerative spine diseases did not vary significantly with age (32).

CCI, which assesses patients' general health status in terms of comorbidities (23, 45), was first introduced in this study to determine factors associated with depression and anxiety in CDDD patients. Results of our study suggested that patients with a higher CCI score had a higher risk of developing depression and anxiety; however, it was not an independent risk factor. The bivariate correlation analysis showed that CCI score was significantly associated with mJOA score (Table 2), the latter of which was a strong and stable independent predictive factor for depression and anxiety (Table 3). Moreover, symptom duration, symptomatology, and surgery history were also significantly associated with mJOA score. This indicated that patients with high CCI, long symptom duration, myelopathy or myeloradiculopathy symptoms, and revision surgery had more severe spinal cord dysfunction. Clinically, the mJOA score can be considered a reflection of the combined effects of these factors. However, the effects of these factors on depression and anxiety may be statistically attributed to the mJOA score; thus, they were excluded following the stepwise regression analysis.

This study has several limitations. First, our study design did not allow cause-effect conclusions. Although cross-sectional observational studies cannot determine causality between variables, associations between demographic and clinical characteristics and depression/anxiety can be confirmed using rigorous statistical analysis (46, 47). Indeed, depression and anxiety often coexist with neck pain or spinal cord dysfunction, and their effects interact with each other (5, 48). Second, depression and anxiety status were assessed using screening scales rather than diagnosed by a specialist in psychological medicine, which is the case in numerous previous studies (13, 34, 35, 39, 40, 46, 47, 49). However, given that SDS and SAS have been validated and are widely accepted screening tools for assessing depression and anxiety, respectively (16), the mental health status determined by these assessment scales can be deemed reliable in this study. Third, the present study investigated the factors associated with depression and anxiety in CDDD patients based on single-center experiences. There is the possibility of selection and indication bias given the study design. Fourth, although the interaction between variables has been taken into consideration, its potential influence on results cannot be completely adjusted by the statistical methods in this study. Thus, the effect of independent risk factors on depression/anxiety may be overestimated. Fifth, it is hard to fully include all potential factors that may influence the incidence of depression and anxiety. The absence of these factors, such as refractive error (50, 51) and menopausal women (52, 53), may affect the evaluation of the effect value. Despite these limitations, this is the first report to expressly point out which patients with CDDD are susceptible to depression and anxiety based on the cross-sectional case-control study design.

## Conclusion

About one-third of CDDD patients have depression or anxiety. Patients who engaged in physical work and had severe symptoms (poor mJOA and high VAS-neck scores) are susceptible to depression and anxiety. Additionally, female patients are more susceptible to depression than man. More attention should be paid to CDDD patients with higher susceptibility to depression or anxiety. Our findings may help identify CDDD patients with depression and anxiety in clinical practice, leading to detailed psychological assessment and appropriate interventions in these patients before cervical spine surgery.

## Data availability statement

The original contributions presented in the study are included in the article/supplementary material, further inquiries can be directed to the corresponding author.

## Ethics statement

The studies involving human participants were reviewed and approved by Ethics Committee of Beijing Tiantan Hospital, Capital Medical University. The patients/participants provided their written informed consent to participate in this study.

## Author contributions

DS and BL were involved in the conception and study design. BX, JZ, and YZ were responsible for data collection. DS, TR, and BW were involved in the writing and revision of the manuscript. DS and WC were responsible for the data analysis. All authors were responsible for critical revision of the manuscript. All authors contributed to the article and approved the submitted version.

## References

[1] Alvin MD Qureshi S Klineberg E Riew KD Fischer DJ Norvell DC. Cervical degenerative disease: systematic review of economic analyses. Spine. (2014) 39(22 Suppl. 1):S53–64. 10.1097/BRS.0000000000000547 25299260

[2] Kazeminasab S Nejadghaderi SA Amiri P Pourfathi H Araj-Khodaei M Sullman MJM. Neck pain: global epidemiology, trends and risk factors. BMC Musculoskelet Disord. (2022) 23:26. 10.1186/s12891-021-04957-4 34980079 PMC8725362

[3] Haldeman S Carroll L Cassidy JD. Findings from the bone and joint decade 2000 to 2010 task force on neck pain and its associated disorders. J Occup Environ Med. (2010) 52:424–7. 10.1097/JOM.0b013e3181d44f3b 20357682

[4] Moradi-Lakeh M Forouzanfar MH Vollset SE El Bcheraoui C Daoud F Afshin A. Burden of musculoskeletal disorders in the Eastern Mediterranean Region, 1990-2013: findings from the global burden of disease study 2013. Ann Rheum Dis. (2017) 76:1365–73. 10.2337/dc16-1075 28209629 PMC5738600

[5] Liu F Fang T Zhou F Zhao M Chen M You J. Association of depression/anxiety symptoms with neck pain: a systematic review and meta-analysis of literature in China. Pain Res Manag. (2018) 2018:3259431. 10.1155/2018/3259431 30356353 PMC6176305

[6] Disease GBD Injury I Prevalence C. Global, regional, and national incidence, prevalence, and years lived with disability for 310 diseases and injuries, 1990-2015: a systematic analysis for the global burden of disease study 2015. Lancet. (2016) 388:1545–602. 10.1016/S0140-6736(16)31678-6 27733282 PMC5055577

[7] Miyamoto GC Lin CC Cabral CMN van Dongen JM van Tulder MW. Cost-effectiveness of exercise therapy in the treatment of non-specific neck pain and low back pain: a systematic review with meta-analysis. Br J Sports Med. (2019) 53:172–81. 10.1136/bjsports-2017-098765 29678893

[8] Fehlings MG Cho S Mroz T Harrop JS Shamji MF Kim J. State of the art in degenerative cervical myelopathy: an update on current clinical evidence. Neurosurgery. (2017) 80:S33–45. 10.1093/neuros/nyw083 28350949

[9] Karpova A Arun R Davis AM Kulkarni AV Massicotte EM Mikulis DJ. Predictors of surgical outcome in cervical spondylotic myelopathy. Spine. (2013) 38:392–400. 10.1097/BRS.0b013e3182715bc3 23448898

[10] Tetreault LA Karpova A Fehlings MG. Predictors of outcome in patients with degenerative cervical spondylotic myelopathy undergoing surgical treatment: results of a systematic review. Eur Spine J. (2015) 24 (Suppl. 2):236–51. 10.1007/s00586-013-2658-z 23386279

[11] Diebo BG Tishelman JC Horn S Poorman GW Jalai C Segreto FA. The impact of mental health on patient-reported outcomes in cervical radiculopathy or myelopathy surgery. J Clin Neurosci. (2018) 54:102–8. 10.1016/j.jocn.2018.06.014 29907392

[12] MacDowall A Skeppholm M Lindhagen L Robinson Y Olerud C. Effects of preoperative mental distress versus surgical modality, arthroplasty, or fusion on long-term outcome in patients with cervical radiculopathy. J Neurosurg Spine. (2018) 29:371–9. 10.3171/2018.2.SPINE171378 30004317

[13] Skeppholm M Fransson R Hammar M Olerud C. The association between preoperative mental distress and patient-reported outcome measures in patients treated surgically for cervical radiculopathy. Spine J. (2017) 17:790–8. 10.1016/j.spinee.2016.02.037 27016269

[14] Tetreault L Nagoshi N Nakashima H Singh A Kopjar B Arnold P. Impact of depression and bipolar disorders on functional and quality of life outcomes in patients undergoing surgery for degenerative cervical myelopathy: analysis of a combined prospective dataset. Spine. (2017) 42:372–8. 10.1097/BRS.0000000000001777 27398891

[15] Alvin MD Miller JA Lubelski D Nowacki AS Scheman J Mathews M. The impact of preoperative depression and health state on quality-of-life outcomes after anterior cervical diskectomy and fusion. Global Spine J. (2016) 6:306–13. 10.1055/s-0035-1562932 27190731 PMC4868578

[16] Li S Qi M Yuan W Chen H. The impact of the depression and anxiety on prognosis of cervical total disc replacement. Spine. (2015) 40:E266–71. 10.1097/BRS.0000000000000743 25494313

[17] Alvin MD Miller JA Sundar S Lockwood M Lubelski D Nowacki AS. The impact of preoperative depression on quality of life outcomes after posterior cervical fusion. Spine J. (2015) 15:79–85. 10.1016/j.spinee.2014.07.001 25016188

[18] Stoffman MR Roberts MS King JT Jr. Cervical spondylotic myelopathy, depression, and anxiety: a cohort analysis of 89 patients. Neurosurgery. (2005) 57:307–13; discussion 13. 10.1227/01.NEU.0000166664.19662.43 16094160

[19] Diebo BG Lavian JD Liu S Shah NV Murray DP Beyer GA. The impact of comorbid mental health disorders on complications following cervical spine surgery with minimum 2-year surveillance. Spine. (2018) 43:1455–62. 10.1097/BRS.0000000000002651 29579013

[20] Zielinski G Ginszt M Zawadka M Rutkowska K Podstawka Z Szkutnik J. The relationship between stress and masticatory muscle activity in female students. J Clin Med. (2021) 10:3459. 10.3390/jcm10163459 34441752 PMC8397028

[21] Jablonska R Slusarz R Krolikowska A Haor B Antczak A Szewczyk M. Depression, social factors, and pain perception before and after surgery for lumbar and cervical degenerative vertebral disc disease. J Pain Res. (2017) 10:89–99. 10.2147/JPR.S121328 28115868 PMC5222600

[22] Theodore N. Degenerative cervical spondylosis. N Engl J Med. (2020) 383:159–68. 10.1056/NEJMra2003558 32640134

[23] Radcliff K Ong KL Lovald S Lau E Kurd M. Cervical spine surgery complications and risks in the elderly. Spine. (2017) 42:E347–54. 10.1097/BRS.0000000000001799 28291765

[24] Kalsi-Ryan S Singh A Massicotte EM Arnold PM Brodke DS Norvell DC. Ancillary outcome measures for assessment of individuals with cervical spondylotic myelopathy. Spine. (2013) 38(22 Suppl. 1):S111–22. 10.1097/BRS.0b013e3182a7f499 23963009

[25] Tetreault L Kopjar B Nouri A Arnold P Barbagallo G Bartels R. The modified Japanese orthopaedic association scale: establishing criteria for mild, moderate and severe impairment in patients with degenerative cervical myelopathy. Eur Spine J. (2017) 26:78–84. 10.1007/s00586-016-4660-8 27342612

[26] Khan I Sivaganesan A Archer KR Bydon M McGirt MJ Nian H. Does neck disability index correlate with 12-month satisfaction after elective surgery for cervical radiculopathy? Results from a national spine registry. Neurosurgery. (2020) 86:736–41. 10.1093/neuros/nyz231 31268151

[27] Richardson SS Berven S. The development of a model for translation of the neck disability index to utility scores for cost-utility analysis in cervical disorders. Spine J. (2012) 12:55–62. 10.1016/j.spinee.2011.12.002 22209244

[28] Zung WW. A self-rating depression scale. Arch Gen Psychiatry. (1965) 12:63–70. 10.1001/archpsyc.1965.01720310065008 14221692

[29] Zung WW. A rating instrument for anxiety disorders. Psychosomatics. (1971) 12:371–9. 10.1016/S0033-3182(71)71479-0 5172928

[30] D'Angelo C Mirijello A Ferrulli A Leggio L Berardi A Icolaro N. Role of trait anxiety in persistent radicular pain after surgery for lumbar disc herniation: a 1-year longitudinal study. Neurosurgery. (2010) 67:265–71. 10.1227/01.NEU.0000371971.51755.1C 20644411

[31] Lobner M Luppa M Matschinger H Konnopka A Meisel HJ Gunther L. The course of depression and anxiety in patients undergoing disc surgery: a longitudinal observational study. J Psychosom Res. (2012) 72:185–94. 10.1016/j.jpsychores.2011.10.007 22325697

[32] Chen Z Luo R Yang Y Xiang Z. The prevalence of depression in degenerative spine disease patients: a systematic review and meta-analysis. Eur Spine J. (2021) 30:3417–27. 10.1007/s00586-021-06977-z 34476597

[33] Levin JM Rabah NM Winkelman RD Mroz TE Steinmetz MP. The impact of preoperative depression on hospital consumer assessment of healthcare providers and systems survey results in a cervical spine surgery setting. Spine. (2020) 45:65–70. 10.1097/BRS.0000000000003222 31513099

[34] Rahman R Ibaseta A Reidler JS Andrade NS Skolasky RL Riley LH. Changes in patients' depression and anxiety associated with changes in patient-reported outcomes after spine surgery. J Neurosurg Spine. (2020). 10.3171/2019.11.SPINE19586. [Epub ahead of print]. 32005017

[35] Doi T Nakamoto H Nakajima K Hirai S Sato Y Kato S. Effect of depression and anxiety on health-related quality of life outcomes and patient satisfaction after surgery for cervical compressive myelopathy. J Neurosurg Spine. (2019). 10.3171/2019.6.SPINE19569. [Epub ahead of print]. 31518976

[36] Kessler RC Berglund P Demler O Jin R Koretz D Merikangas KR. The epidemiology of major depressive disorder: results from the national comorbidity survey replication (NCS-R). JAMA. (2003) 289:3095–105. 10.1001/jama.289.23.3095 12813115

[37] de Graaf R ten Have M van Gool C van Dorsselaer S. Prevalence of mental disorders and trends from 1996 to 2009. Results from the Netherlands mental health survey and incidence study-2. Soc Psychiatry Psychiatr Epidemiol. (2012) 47:203–13. 10.1007/s00127-010-0334-8 21197531

[38] Baxter AJ Scott KM Vos T Whiteford HA. Global prevalence of anxiety disorders: a systematic review and meta-regression. Psychol Med. (2013) 43:897–910. 10.1017/S003329171200147X 22781489

[39] Divi SN Goyal DKC Mangan JJ Galetta MS Nicholson KJ Fang T. Are outcomes of anterior cervical discectomy and fusion influenced by presurgical depression symptoms on the mental component score of the short form-12 survey? Spine. (2020) 45:201–7. 10.1097/BRS.0000000000003231 31513106

[40] Goh GS Liow MHL Yeo W Ling ZM Guo CM Yue WM. Poor baseline mental health does not influence improvement in patient-reported outcomes, satisfaction, and return to work two years after single-level anterior cervical discectomy and fusion. Spine. (2019) 44:839–47. 10.1097/BRS.0000000000002960 30540718

[41] Gagné S Vasiliadis HM Préville M. Gender differences in general and specialty outpatient mental health service use for depression. BMC Psychiatry. (2014) 14:135. 10.1186/1471-244X-14-135 24884891 PMC4028012

[42] Jacobi F Wittchen HU Holting C Hofler M Pfister H Muller N. Prevalence, co-morbidity and correlates of mental disorders in the general population: results from the German health interview and examination survey (GHS). Psychol Med. (2004) 34:597–611. 10.1017/S0033291703001399 15099415

[43] Kuehner C. Why is depression more common among women than among men? Lancet Psychiatry. (2017) 4:146–58. 10.1016/S2215-0366(16)30263-2 27856392

[44] Strom J Bjerrum MB Nielsen CV Thisted CN Nielsen TL Laursen M. Anxiety and depression in spine surgery-a systematic integrative review. Spine J. (2018) 18:1272–85. 10.1016/j.spinee.2018.03.017 29649613

[45] Charlson ME Pompei P Ales KL MacKenzie CR. A new method of classifying prognostic comorbidity in longitudinal studies: development and validation. J Chronic Dis. (1987) 40:373–83. 10.1016/0021-9681(87)90171-8 3558716

[46] Wu M Shen L Wang Q Liu L Lu S Jin J. Anxiety and depression prevalence and risk factors among patients with cardiovascular diseases in post-COVID-19 China. Front Public Health. (2021) 9:758874. 10.3389/fpubh.2021.758874 35059375 PMC8763782

[47] Borie YA Siyoum M Tsega A Anbese G. Maternal depression and associated factors among pregnant women attending ante natal care, southern ethiopia: cross-sectional study. Front Public Health. (2022) 10:848909. 10.3389/fpubh.2022.848909 35757655 PMC9223634

[48] Lin SY Sung FC Lin CL Chou LW Hsu CY Kao CH. Association of depression and cervical spondylosis: a nationwide retrospective propensity score-matched cohort study. J Clin Med. (2018) 7:387. 10.3390/jcm7110387 30366474 PMC6262285

[49] Yoo JS Jenkins NW Parrish JM Brundage TS Hrynewycz NM Mogilevsky FA. Evaluation of postoperative mental health outcomes in patients based on patient-reported outcome measurement information system physical function following anterior cervical discectomy and fusion. Neurospine. (2020) 17:184–9. 10.14245/ns.1938256.128 32054139 PMC7136091

[50] Zhang H Gao H Zhu Y Zhu Y Dang W Wei R. Relationship between myopia and other risk factors with anxiety and depression among Chinese university freshmen during the COVID-19 pandemic. Front Public Health. (2021) 9:774237. 10.3389/fpubh.2021.774237 34926391 PMC8671746

[51] Wu Y Ma Q Sun HP Xu Y Niu ME Pan CW. Myopia and depressive symptoms among older Chinese adults. PLoS ONE. (2017) 12:e0177613. 10.1371/journal.pone.0177613 28498851 PMC5428930

[52] Soares CN. Depression in peri- and postmenopausal women: prevalence, pathophysiology and pharmacological management. Drugs Aging. (2013) 30:677–85. 10.1007/s40266-013-0100-1 23801148

[53] Bromberger JT Kravitz HM Chang Y Randolph JF Jr., Avis NE, et al. Does risk for anxiety increase during the menopausal transition? Study of women's health across the nation. Menopause. (2013) 20:488–95. 10.1097/gme.0b013e3182730599 23615639 PMC3641149

